# Health Effects of Desert Dust Storms in Children With Asthma: Knowledge, Perceptions and Practices of School Health Visitors in Cyprus

**DOI:** 10.1155/nrp/8840328

**Published:** 2025-04-16

**Authors:** Paraskevi Kinni, Panayiotis Kouis, Melanie Charalambous, Maria G. Kakkoura, Stavria-Artemis Elia, Eirini Kampriani, Souzana Achilleos, Andrie Panayiotou, Maria Hadjisoteriou, Nicos Middleton, Panayiotis K. Yiallouros

**Affiliations:** ^1^Respiratory Physiology Laboratory, Medical School, University of Cyprus, Nicosia, Cyprus; ^2^Cyprus International Institute for Environmental & Public Health, School of Health Sciences, Cyprus University of Technology, Limassol, Cyprus; ^3^Department of Nursing, School of Health Sciences, Cyprus University of Technology, Limassol, Cyprus; ^4^Educational Sector, Nursing Services, Ministry of Health, Nicosia, Cyprus; ^5^Clinical Trial Service Unit and Epidemiological Studies Unit, Department of Population Health, University of Oxford, Oxford, UK; ^6^Department of Social and Political Sciences, University of Cyprus, Nicosia, Cyprus; ^7^Department of Basic and Clinical Sciences, Medical School, University of Nicosia, Nicosia, Cyprus; ^8^Department of Primary Care and Population Health, Medical School, University of Nicosia, Nicosia, Cyprus; ^9^School Health Services, Ministry of Health, Nicosia, Cyprus

**Keywords:** air pollution, asthma, desert dust storms, school health services, school health visitors

## Abstract

**Introduction:** Cyprus is located in the Eastern Mediterranean and is heavily exposed to desert dust storm (DDS) events, which diminish air quality and adversely impact paediatric asthma morbidity. School health visitors (SHVs) play a key role in asthma management in schoolchildren and are pivotal for the development of school-based intervention programs.

**Objectives:** This study aimed to examine the knowledge, practices and perceptions of SHVs on paediatric asthma management. Additionally, we sought to determine the factors associated with SHVs' level of engagement in school-based intervention programs to mitigate DDS-associated health effects.

**Methods:** An anonymous questionnaire on asthma management practices and perceptions was administered to all SHVs in Cyprus. We assessed the association between the perceived importance and real-life implementation of asthma management practices and, in addition, asthma management practices and preparedness of local authorities to respond to DDS events were compared across categories for different sociodemographic characteristics. A binary logistic regression analysis was carried out to determine predictors among SHVs for supporting a school-based intervention program for DDS.

**Results:** Responses from 78/93 SHVs (84%) revealed mean estimates for perceived importance ranging between 8.20 (lowest) for performing regular check-ups and 9.6 (highest) for monitoring student health records and immunisation status. Significant differences were demonstrated between the perceived importance of most of the reported asthma management practices and the extent of their implementation. Moderate appreciation among SHVs on a 1–5 Likert scale was reported for the regulatory authorities' preparedness (*M* = 3.48, SD = 0,88) and current practices (*M* = 2.66, SD = 0.13) to respond to DDS.

**Conclusion:** Asthma management practices in school settings in Cyprus, a country highly exposed to DDS events, are suboptimal and responses during DDS are fragmented. Despite the perceived low preparedness, from the authorities, SHVs recognise the health impact of DDS on children with asthma and support plans for desert dust-mitigation programs in schools.

## 1. Introduction

Childhood asthma is a growing public health concern worldwide as it is the most common chronic condition in children and one of the leading causes of emergency department visits, hospitalisations and school absenteeism [1]. In areas characterised by high levels of air pollution, children with asthma are exposed to various pollutants such as particulate matter, nitrogen dioxide, ozone, and sulphur dioxide. These pollutants can irritate the airways, cause inflammation and lead to increased respiratory issues among this susceptible group. Similarly, exposure to desert dust storm (DDS) events has been shown to be associated with worsening asthma symptoms, reduced lung function and increased medication purchases [2–4].

The island of Cyprus, located in the Eastern Mediterranean, recorded increased asthma frequency among primary school children between 2000 and 2008 [5] and is exposed to frequent and intense DDS originating from the neighbouring Sahara and Arabian deserts [6, 7]. In recent years, the increased frequency and intensity of DDS events across the Mediterranean basin has been linked with advancing climate change [6, 8, 9], thus fuelling a growing debate in the region to reevaluate current and develop new public health policies in order to protect children with asthma from the growing DDS health risks.

The school health visitors (SHVs) public service employs nursing and medical personnel with the mission to perform health monitoring assessments, screening tests, infectious disease control and promotion of health awareness in school children. SHVs perform regular visits to schools in their area of jurisdiction and remain in frequent communication with students, school personnel and parents. Consequently, SHVs constitute a key component in childhood asthma management within the school environment. As, in many occasions, SHVs have more frequent contact with children compared to other health professionals, and they participate as well as engage the family and school personnel in health education and health promotion activities [10]. As such, SHVs constitute an important target group towards the development of future intervention policies aiming to protect children with asthma from DDS events, especially at the community level. Evidence from psychological and behavioural theory (Health Belief Model) highlights the importance of personal disease and risk factors awareness and the perceived susceptibility to it. Combined with a person's belief in the effectiveness of the recommended interventions or health behaviours, these parameters can predict the likelihood that the person will adopt an intervention or health-protecting behaviour [11]. SHVs, under this theoretical framework, have an important role in educating children (and their families) about asthma, emphasising the importance of medication adherence, recognising early symptoms of asthma exacerbation and understanding the role of environmental triggers such as DDS events. Additionally, SHVs can engage in health promotion activities, such as organising asthma awareness campaigns, and support a variety of school-based intervention programs for DDS and other risk factors [12]. However, to this date, there is a considerable lack of data on the knowledge, perceptions and current practices of SHVs working with children in school settings regarding childhood asthma management practices and the mitigation of the health effects during DDS events.

This study aimed to examine SHVs' knowledge about childhood asthma and current management strategies in Cyprus, with a particular focus on their awareness of the health effects of DDS and relevant management practices. Additionally, the study sought to assess SHVs' perceptions of school-based intervention programs designed to mitigate the health effects of DDS and to identify the factors associated with SHVs' willingness to engage in these programs.

## 2. Materials and Methods

### 2.1. Design and Study Population

A cross-sectional survey was conducted in the spring 2019 to collect data from SHVs currently working in public schools across Cyprus. The survey targeted all currently employed SHVs, regardless of their school area of service, level of education, years of professional experience and years working specifically as an SHV. The questionnaire was completed and collected anonymously in a sealed envelope prior to two full-day workshops organised in May and June 2019. These workshops were designed to discuss the impacts of DDS on human health, and all participants were fully informed about the purpose of this study and provided written informed consent before their participation in the study. The study protocol and questionnaire received ethical approval from the Cyprus National Bioethics Committee (ΕΕΒΚ ΕΠ 2019.01.97).

### 2.2. Study Questionnaire

The structured questionnaire was developed based on a comprehensive literature review and consideration of previously described similar questionnaires [10, 11] and is available (in the English language) as Supporting Information 1. The questionnaire included 50 questions, divided into five sections titled ‘Demographic characteristics', ‘Current practices for management of childhood asthma', ‘Assessment of the preparedness of local authorities to respond to DDS events', ‘Asthma management within the school setting in relation to DDS events', and ‘Perceptions about future school-based interventions during DDS events'. All items were scored on a five-point Likert response scale, except for the item on perceived importance of asthma management practices which was scored on a 1–10 rating scale. Overall, the questionnaire was characterised by high internal consistency and stability, with Cronbach's alpha coefficients for all subscales ranging from 0.81 to 0.96.

### 2.3. Statistical Analysis

Descriptive statistics (counts and percentages) were used to summarise the demographic, educational and professional characteristics of participating SHVs. For each questionnaire item, mean and standard deviation (SD) were calculated, and a summary mean score was calculated for each section of the questionnaire to provide an overall assessment of the responses in the particular area. To evaluate the association between perceived importance for an asthma management practice and the implementation of the corresponding practices, we created for each practice three categories based on perceived importance: ‘*Very Important*' (score of 10), ‘*Important*' (score of 8-9) and ‘*Less Important*' (scores 1–7). These categories were established to facilitate the interpretation of the examined relationships and to reduce the impact of skewness in the perceived importance variables. The categorisation relied on treating the maximum score of 10 as the ideal condition (highest category—‘*Very Important*'), scores 8-9 as the second category (‘Important') and scores 1–7 as the lowest category ‘Less important' as data were sparse at low scores. Similarly, variables on the implementation of asthma management practices were categorised as ‘Less Frequent' (scores 1-2) and ‘More Frequent' (scores 3–5). The chi-square test of independence was used to check whether the distribution of the perceived importance categories was independent of the distribution of the corresponding implementation categories. Differences in summary scores for childhood asthma management and for the assessment of the preparedness of local authorities to respond to DDS events across different sociodemographic groups were assessed using analysis of variance (ANOVA). To account for the risk of Type I error due to multiple comparisons, we applied the Bonferroni correction in all analyses.

Finally, a binary logistic regression analysis was performed for the identification of predictors among SHVs for supporting a school-based intervention program for DDS events. For the purposes of this study, the overall support of SHVs for a school-based intervention program was measured with questionnaire item 36 (measured on a five-point Likert Scale) that was converted into two categories: ‘Less Support' (scores 2, 3 and 4) treated as the reference category and ‘More Support' (score of 5), based on the median value to reduce the impact of skewness. The logistic regression analyses were performed in three steps, a univariable regression, a multivariable regression and then a backward stepwise logistic regression. The results of the logistic regression analyses are presented as odds ratios (ORs) with 95% confidence interval. All statistical analyses were carried out using IBM SPSS Statistics 25, and *p* values of < 0.05 were considered statistically significant.

## 3. Results

### 3.1. Sample Characteristics

Of the 93 SHVs that were invited to participate in the two workshops, 78 completed the questionnaire.  Table 1 presents the participants' demographic and professional characteristics. The sample was homogeneous in terms of gender, as the great majority of SHVs were females (95.7%), and most of them (75.3%) were aged 30–49 years. Their mean professional experience as a nurse was 16.5 (SD = 7.9) years, while the mean experience as an SHV was 13.5 (SD = 7.3) years. Nevertheless, only 23.4% were working full time in the school setting, with many (68.8%) sharing their working hours between schools and other community settings (e.g., maternity and child welfare clinics, municipal health care centres and community palliative services). Almost half (45.5%) of the SHVs had a postgraduate degree, while another 46.8% held a BSc degree. Three-quarters of the participants (75.4%) reported that they were assigned the health monitoring of > 1000 students.

### 3.2. Research Question Results

#### 3.2.1. Practices Towards Childhood Asthma Management

In Table 2, we present the frequency of childhood asthma management practices performed by SHVs within the school environment. The practices that received the highest score (i.e., performed more frequently) were as follows: (1) access to data on children's health provided by the schools' administration (e.g., information on which children have asthma and their medication status), (2) updated health records and immunisation status and (3) communication with parents' of the children with asthma regarding laboratory investigations, medication regimen and disease course. In contrast, the practices that received the lowest scores (i.e., not reported to occur frequently) were as follows: tracking of missed schooling due to asthma-related symptoms, assessment of asthma control and level of awareness of asthma triggers.

#### 3.2.2. Rating of the Importance of Childhood Asthma Management Practices

**Figure 1:**
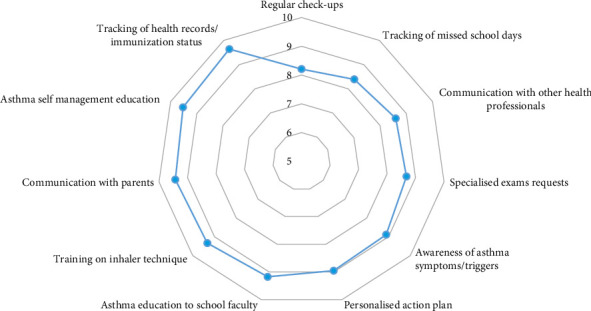
SHV perceived importance (scale: 1–10) of current practices for childhood asthma management within the school environment.

The majority of responders rated all the above as practices of high importance, regardless of whether these were implemented in everyday practice or not. On a 1–10 scale, mean estimates for perceived importance ranged between 8.20 (lowest) for performing regular check-ups and 9.6 (highest) for monitoring student health records and immunisation status (Figure 1).

Chi-square test was performed to examine the association between the perceived importance and the actual level of implementation of specific practices for asthma management. The communication of laboratory investigation results and referring students with poor control to an asthma specialist yielded statistically significant associations (Table 3).

#### 3.2.3. Assessment of Preparedness to Respond to DDS Events

The mean summary score in this section was 3.48 (SD = 0.88), indicating moderate appreciation among SHVs of the regulatory authorities' preparedness to respond to DDS (Table 4). The majority of SHVs seem to be aware of the adverse health effects of DDS in children with asthma (mean = 4.74, SD = 0.51) and also recognise that climate change will further increase the health-related problems among schoolchildren (mean = 4.69, SD = 0.62). For items that directly evaluated the authorities' preparedness to mitigate the impacts of DDS events, the mean rating was lower than 3.5. According to the SHV assessment, the Ministry of Health is more aware of the potential effects of DDS events on children's health and has sufficient experience to develop an effective plan to mitigate these effects, compared to the Ministry of Education or other governmental bodies.

#### 3.2.4. Current Practices Implemented in Relation to DDS Events

The mean evaluation by SHVs of the current practices of regulatory authorities in response to DDS events was 2.66 (SD = 0.13) on a 1–5 Likert scale. The most highly graded practice was keeping records of children with asthma and schools' adherence to measures issued by the Ministry of Education during DDS episodes which yielded average scores > 3.8. Most notably, SHVs considered that regulatory authorities underutilise the official ‘Air Quality Cyprus' internet application (https://www.airquality.dli.mlsi.gov.cy/), which provides real-time information on air quality in Cyprus (mean = 1.40, SD = 0.10). Other items, such as whether the school health service propagates warnings and guidelines about DDS events, as well as whether regulatory authorities organise and implement information campaigns within the school environment, also received low scores (< 2.5 on a 1–5 Likert scale) (Table 5).

ANOVA demonstrated a statistically significant association between work experience as an SHV and administrative district. Both mean scores on asthma management practices and evaluation of the preparedness of local regulatory authorities to respond to DDS were significantly lower in the group with working experience as an SHV between 11 and 20 years compared to those with working experience as an SHV of less than 10 years. Similarly, we observed significant discrepancies in the mean scores for asthma management practices and assessment of the current practices of regulatory authorities between SHVs from the peripheral districts of Limassol and Paphos and the SHVs from the central district of Nicosia (Table 6).

#### 3.2.5. Determinants of Support for a School-Based Intervention Program for DDS Events

Table 7 presents the association between SHVs characteristics and the overall support to a school-based intervention program for DDS events. The analysis included factors such as education level, working experience as a nurse, and working experience as an SHV, as well as awareness of DDS-related health effects, the total score of SHV asthma management practices, and the total score for current practices of SHV service to respond to DDS events. In the univariate logistic analysis, SHVs who stated that DDS events may have adverse effects on children with asthma were 7-8 times more likely to support a school-based intervention program for DDS events. Furthermore, SHVs who supported the statement that climate change will further increase DDS-related health effects among children are 3 times more likely to support a school-based intervention program. However, in the multivariate binary stepwise logistic regression analysis, only the perception that DDS episodes may have possible adverse effects on children with asthma was found to be independently related and predictive of support of a school-based intervention program [OR = 7.33 (2.12–25.31) *p* < 0.001].

## 4. Discussion

This is the first study worldwide that explored the practices and perceptions of SHVs in relation to DDS events and their effects on schoolchildren with asthma and was conducted towards developing evidence-based recommendations to reduce exposure to desert dust, as part of an adaptation strategy for DDS events in South-eastern Europe. Our findings demonstrate that asthma management in school settings in Cyprus is suboptimal with important elements such as tracking school absenteeism, assessing asthma control and monitoring awareness of asthma triggers, being poorly or not consistently implemented. This suboptimal practice was reported despite the acknowledgement by SHVs that these aspects of asthma management are important, thus highlighting gaps in practice despite the perceived necessity. Furthermore, although SHVs recognise the health impact of DDS on children with asthma, the perceived preparedness of regulatory authorities to respond to DDS as well as the current level of disseminating warnings or providing information for DDS in the school setting was considered low to moderate. Nevertheless, SHVs, and especially SHVs who were more aware of the potential health effects of DDS and climate change, demonstrated high levels of support for school-based intervention programs for DDS events, highlighting their potential role as a key professional group in implementing policies that aim to mitigate the effects of air pollution and climate change stressors on children's health.

SHVs in Cyprus have an important role in monitoring the health status of schoolchildren with asthma, and they may contribute to their management by providing counselling and education to them, their families and school staff [13]. Their role is evidently more important in regions with increased exposure to DDS events and other climate change–related stressors. SHVs may also be pivotal in identifying social or other factors that increase the susceptibility of children with asthma to desert dust or anthropogenic air pollution [14] and in educating parents and children alike for the health effects of this phenomenon, as well as educating school personnel on the importance of maintaining good indoor air quality [15]. During DDS events, they can monitor symptoms among students at risk and ensure adherence to medication, as well as coordinate care with families and healthcare providers [16].

Our study also demonstrated that asthma management practices within the school environment in a country heavily impacted by DDS like Cyprus are currently suboptimal. Especially, in relation to tracking school absenteeism and assessment of asthma control, SHVs can have an important role. Previous studies have provided evidence that desert dust is linked to absence from school due to sickness and increased symptoms or hospitalisations of children with asthma [3, 17–19]. In this context, tracking of missed school days and assessment of asthma control have been found to be key elements of successful nurse-led programs focusing on children with asthma, such as the ‘Building Bridges for Asthma Care' [20] and ‘Easy Breathing for Schools' [21] programs.

Our results demonstrated significant discrepancies between the perceived importance of various asthma management practices and the actual extent of their implementation by SHV services. Previous studies have examined in detail the challenges and barriers faced by the nursing personnel in schools across different countries and their association with lack of resources, time constraints, communication challenges (between healthcare professionals, parents and teachers), limited knowledge and lack of awareness [22–24]. Although not directly assessed in our study, it is possible that similar barriers may exist in Cyprus, which resulted in moderate associations between the perceived importance and actual extent of implementation of relevant asthma management practices in the school environment. In addition, SHV service in Cyprus is further limited by the large number of students under their care that is usually spread out across different schools. Previous reports highlighted the importance of these factors and demonstrated that high school nurse/student ratios and allocation of duties to nurses in more than one school results in limited communication with school professionals, students and parents, worsening of time constraints and confusion in SHVs' responsibilities [25]. In this context, schools tend to provide poor quality services to children with asthma [26], while an increase in the time school nurses spend within the school environment leads to improved asthma management [27].

### 4.1. Strengths and Limitations

The study followed a quantitative study design and made use of a specifically designed questionnaire aiming to capture school-based asthma management practices, as well as the knowledge and perceptions of SHVs in relation to DDS events and school-based intervention programs aiming to reduce exposure to air pollution. As the participation rate in the study was particularly high (84%), our results are representative of the knowledge, practices and perceptions of the target population. Of course, the differences in educational and school health systems may reduce the generalisability of our findings to other country settings, but our findings are expected to be of particular interest to countries affected by desert dust. In these countries, increased awareness among SHVs, other healthcare professionals and stakeholders can lead to action plans for asthma management that consider air pollution and DDS events. In addition, data were collected prior to the onset of the COVID-19 pandemic and more recent data are not available for comparison, particularly considering potential shifts in asthma management practices. Nevertheless, the risk perception for environmental health risks such as air pollution has not been reduced in the postpandemic era [28, 29], while the significance of the school environment for public health policy [30, 31], as well as the critical role of SHVs or nurses for health promotion, was further enhanced during the pandemic [32, 33]. As such, although these data were collected in the prepandemic era, the relevance of our findings remains high. Furthermore, as responders were participants in workshops related to the subject of the study and to mitigate any risk of bias arising from attending the workshops, the questionnaires were administered before the workshops took place.

### 4.2. Implications for Practice

In countries affected by Saharan and Asian desert dust, a higher risk for hospitalisations for childhood asthma exacerbations during desert dust days has been demonstrated repeatedly [2, 3, 34]. These effects are similar to the effects of days with high levels of air pollutants from anthropogenic sources on hospitalisations of children with asthma, outpatient visits, asthma-related missed schooling days and increased antiasthma medication use [35–37]. As children may spend more than 200 days per year in the school environment [38], school-based intervention programs have been proposed towards reducing exposure to air pollution and related respiratory morbidity. These programs have the advantage of simultaneously targeting many children with asthma, in contrast to home-based interventions that benefit only individual families. Possible school-based programs range from interventions to motivate students to use less polluted routes from home to school [39], to replacement of school heaters to reduce indoor emissions [40], improvements in school air filtration infrastructure [41] and installation of classroom-based air purifiers [42, 43]. The latter are also applicable for school-based interventions in countries affected by desert dust like Cyprus [44] and other southern European countries. In Cyprus and Greece, recommendations to reduce outdoor physical activity and the use of air purifiers demonstrated promising results in reducing air pollution and desert dust exposure in children with asthma [45, 46].

Nevertheless, to successfully implement school-based programs, several challenges need to be overcome such as the commitment of senior school administrators, awareness of the health problem targeted, coordination of school visits by healthcare professionals and good communication with parents of children with asthma [47]. SHVs and school nurses can play a key role in overcoming many of these challenges. The engagement of school nurses may facilitate communication between the child, the family, clinicians and the community [48]. Previous studies on the role of school nurses towards the implementation of a school-based asthma intervention highlighted the importance of this first-line group of healthcare professionals in monitoring personalised asthma management action plans and adherence to prescribed medication. However, limitations in time availability and funding may compromise the replicability of these approaches in a larger scale [49, 50].

Our results also provide useful insights into the preparedness of local authorities to respond to DDS events and implement policies aiming at protecting children with asthma. The reported moderate appreciation is in accordance with the gaps in knowledge and practices reported recently by local regulatory authorities and stakeholders in the Eastern Mediterranean region in relation to DDS events and their health effects [51]. Limited implementation of predefined action plans for DDS events and lack of an efficient early alert system and standardised recommendations for exposure reduction among susceptible groups were identified in that report. Similar gaps characterised by absence of DDS-related policies were also described by Allahbakhshi et al. (2019) in the Eastern Mediterranean region [52]. A good working relationship between school nurses and other stakeholders is essential, and the interventions designed to reduce environmental exposures in schools must engage multiple stakeholders, taking into consideration the unique challenges of the school environment and school year timetable [38]. Furthermore, the findings of our study largely indicated that SHVs have a good understanding of the problems, acknowledge the importance of asthma management and are already in support of school-based interventions for asthma. On the other hand, gaps in the implementation of asthma management practices as well as fragmented responses to DDS events from regulatory authorities were also highlighted. To bridge these gaps and enhance an evidence-based and standardised response to DDS in the school environment, an asthma-specific toolkit to assist SHVs in providing support for students with asthma can be made available. Previous examples of similar toolkits have yielded positive results [53–56]. In this context, an adaptation of previous toolkits for the Cyprus school setting should be possible, especially with the involvement of SHVs as the end users. In parallel, the participation of SHVs in policy planning should be further enhanced. As end users or facilitators of public health policy at the school setting, their first-line experiences with affected students and their views on feasibility considerations can ensure that policies are practical, realistic and can be effectively implemented. Within this framework, SHVs can promote and implement school education programs and practices, track and reduce public health risks in school communities, including those related to air pollution and DDS events [57].

## 5. Conclusions

This study demonstrated that SHVs in Cyprus acknowledge that asthma management practices in relation to DDS within the school environment are lacking, although the perceived importance of implementing such good practices is relatively high. SHVs are largely aware of the DDS phenomenon and the related health effects, while they are in support of implementing a school-based intervention program targeting children with asthma. The findings of this study highlight the need for future policy initiatives to support the real-life implementation of more rigorous asthma management practices in the school setting including a school-based intervention program for environmental triggers such as DDS events.

## Figures and Tables

**Table 1:** Demographic and working conditions' characteristics of study participants.

Parameter	Number of subjects (*n*)	Percentage (%)
Enrolment (*n* = 93)	78	83.8

*Sex (n = 69)*		
Women	66	95.7
Men	3	4.3

*Age (n = 77)*		
≤ 29 years	2	2.6
30–39 years	27	35.1
40–49 years	31	40.3
50–59 years	15	19.5
≥ 60 years	2	2.6

*Highest level of education (n = 77)*		
Diploma	1	1.3
Degree	36	46.8
Medical degree	4	5.2
Master	35	45.5
PhD	1	1.3

*Work setting (n = 77)*		
School	18	23.4
School and other setting	59	76.6

*Level of schools under care (n = 68)*		
Primary education	7	10.3
Primary and secondary education	60	88.2
Secondary education	1	1.5

*Total students under care (n = 57)*		
1–500 students	3	5.3
501–1000 students	11	19.3
1001–1500 students	41	71.9
> 1500 students	2	3.51
Working experience as a nurse in years^∗^	16.5	7.9
Working experience as SHV in years^∗^	13.5	7.3

^∗^Mean and standard deviation.

**Table 2:** SHV current practices for childhood asthma management.

Survey questions on current practices for childhood asthma management in descending order in terms of frequency of implementation	Mean^∗^	SD
1. Request updated student health records and monitoring of students' immunisation status.	4.89	0.54
2. Acquisition of information from school on children with asthma as well as their medication status at the beginning of the school year.	4.31	1.21
3. Acquisition of information from parents of children with asthma for laboratory investigation results, medication and course of disease.	4.03	1.01
4. Referring students to primary health care if their asthma is not under control.	3.74	1.30
5. Acquisition of information from teachers about exacerbations in children with asthma.	3.47	1.04
6. Assessment of implementation of asthma management recommendations by children with asthma and their family.	3.08	1.05
7. Contact health care provider to obtain investigations' results (e.g., peak flow measurements) or management guidelines at the beginning of the school year.	2.80	1.30
8. Assessment of inhalers' use technique of the students with asthma.	2.80	1.15
9. Development of an asthma action plan for each student with asthma.	2.68	1.41
10. Provision of education to students to self-manage their asthma	2.64	1.26
11. Provision of written instructions for asthma management during school hours.	2.61	1.36
12. Assessment of students' level of asthma control (clinical examination) during the school year.	2.42	1.50
13. Tracking missed schooling days for asthma related symptoms in children with asthma.	2.09	1.01
14. Assessment of student's asthma control (symptoms) and awareness of asthma triggers.	1.90	1.40

Abbreviation: SD, standard deviation.

^∗^Responses were coded on a five-point Likert scale: 5 = *always*; 4 = *often*; 3 = *sometimes*; 2 = *rarely*; 1 = *never*.

**Table 3:** Percentage of SHVs performing the asthma management practices according to their rating of the importance of these practices.

Percentage of SHVs performing the practice by importance rating						
Practice		Less important (1–7)	Important (8-9)	Very important (10)	*χ* ^2^	*p* value
Regular check-ups	Less frequent	26.8	36.6	36.6	0.711	0.70
	More frequent	27.6	27.6	44.8		

Awareness of asthma symptoms/triggers	Less frequent	22.2	20.4	57.4	4.618	0.10
	More frequent	0.0	29.4	70.6		

Laboratory investigation requests	Less frequent	29.0	41.9	29.0	8.263	0.02^**∗**^
	More frequent	9.8	29.3	61.0		

Tracking of health records/immunisation status	Less frequent	0.0	100	0.0	4.602	0.10
	More frequent	2.8	16.9	80.3		

Personalised action plan	Less frequent	13.2	28.9	57.9	0.167	0.92
	More frequent	12.5	25.0	62.5		

Asthma self-management education	Less frequent	8.8	14.7	76.5	1.477	0.48
	More frequent	2.5	17.5	80.0		

Communication with parents	Less frequent	18.2	13.6	68.2	1.901	0.38
	More frequent	7.7	19.2	73.1		

Training/assessing inhaler technique	Less frequent	15.4	19.2	65.4	2.812	0.25
	More frequent	4.3	19.1	76.6		

Tracking of missed schooling days	Less frequent	25.5	41.2	33.3	1.596	0.45
	More frequent	13.6	40.9	45.5		

Communication with other health professionals	Less frequent	50.0	21.4	28.6	9.444	0.01^**∗**^
	More frequent	13.3	36.7	50.0		

Asthma education to school faculty	Less frequent	12.5	28.1	59.4	1.949	0.38
	More frequent	5.3	21.1	73.7		

*Note: Less frequent* = score 1-2; *more frequent* = score 3–5.

Abbreviation: SHV, school health visitor.

^∗^ *p* < 0.05 in the chi-square test.

**Table 4:** Appreciation of the preparedness of local authorities to respond to DDS events.

Survey questions on the preparedness of local authorities to respond to DDS events	Mean^∗^	SD
1. DDS episodes may have possible adverse effects on children with asthma.	4.74	0.51
2. Due to climate change, health problems associated with DDS episodes will increase among school-aged children.	4.69	0.62
3. Your service is aware of the potential adverse effects of DDS episodes on children with asthma.	3.39	0.96
4. Ministry of Health is aware of the potential adverse effects of DDS episodes on children with asthma.	3.69	0.87
5. Ministry of Education is aware of the potential adverse effects of DDS episodes on children with asthma.	3.44	0.91
6. Other governmental bodies are aware of the potential adverse effects of DDS episodes on children with asthma.	3.13	0.93
7. Your service currently has sufficient experience to manage the potential health impacts of DDS on school-age children.	3.43	0.89
8. Ministry of Health currently have sufficient experience to manage the potential health impacts of DDS on school-age children.	3.46	0.93
9. Ministry of Education currently have sufficient experience to manage the potential health impacts of DDS on school-age children.	3.01	0.92
10. Other governmental bodies currently have sufficient experience to manage the potential health impacts of DDS on school-age children.	2.76	0.89
11. Your service has the expertise to create an effective plan for adapting to climate change and managing potential health impacts.	3.43	0.89
12. Ministry of Health have the expertise to create an effective plan for adapting to climate change and managing potential health impacts.	3.59	0.92
13. Ministry of Education have the expertise to create an effective plan for adapting to climate change and managing potential health impacts.	3.10	0.88
14. Other governmental bodies have the expertise to create an effective plan for adapting to climate change and managing potential health impacts.	3.01	0.90
15. Managing the impact of DDS episodes on children's health is a priority for your service.	3.49	1.09

Abbreviation: SD, standard deviation.

^∗^Responses were coded on a five-point Likert scale: 5 = *always*; 4 = *often*; 3 = *sometimes*; 2 = *rarely*; 1 = *never*.

**Table 5:** Current practices of school health visitors regarding DDS events.

Questionnaire items^∗^	Mean	SD
1. Your service receives warnings from a responsible government agency when dust storms occur.	1.99	0.15
2. Your service uses the ‘Air Quality Cyprus' application to be informed about the air quality throughout the year.	1.40	0.10
3. Your service manages health issues due to desert dust episodes.	2.43	0.14
4. Instructions are provided by your department for the management of asthma in children during desert dust episodes.	2.43	0.14
5. You receive training/information from your service, through seminars, on new data on the management of childhood asthma during dust storms in the atmosphere.	2.00	0.13
6. During the school year, you keep record of all necessary information for all school children with asthma.	4.21	0.14
7. During DDS dust episodes, children with asthma are monitored by your service.	2.51	0.14
8. During the school year, you give lectures to both teachers and students on topics related to desert dust protection measures.	1.61	0.11
9. During the school year, you disseminate information in the form of informational material with measures to protect from DDS.	1.91	0.13
10. You are aware of any additional desert dust prevention measures taken by other agencies (e.g., Ministry of Education).	3.21	0.13
11. You track missed schooling days of children with asthma due to their asthma exacerbation associated with DDS episodes.	2.81	0.12
12. You get informed for possible asthma exacerbation of children related to DDS episodes from the school stuff.	3.09	0.11
13. Your school is taking preventive measures issued by the Ministry of Education and Culture regarding DDS episodes.	3.85	0.11
14. The school implements and modifies its daily schedule during DDS episodes.	3.81	0.12

Abbreviation: SD, standard deviation.

^∗^Responses were coded on a five-point Likert scale: 5 = *always*; 4 = *often*; 3 = *sometimes*; 2 = *rarely*; 1 = *never*.

**Table 6:** Differences in summary scores for childhood asthma management and appreciation of the preparedness of local authorities to respond to DDS events by sociodemographic characteristics.

		Score for asthma management practices		Score for appreciation of the preparedness of local regulatory authorities to respond to DDS	
		Mean ± SD	*p* value	Mean ± SD	*p* value
Age group	≤ 39	42.4 ± 8.1	0.48	53.8 ± 8.5	0.31
	40–49	43.3 ± 8.4		50.8 ± 8.8	
	≥ 50	39.6 ± 11.3		50.6 ± 7.0	

Education	Graduate degree	41.4 ± 9.7	0.47	51.6 ± 8.0	0.72
	Postgraduate degree	42.9 ± 8.0		52.3 ± 8.9	

Working experience	≤ 10 years	41.2 ± 8.2	0.32	54.5 ± 9.0	0.18
	11–20 years	41.7 ± 7.0		51.4 ± 7.7	
	≥ 21 years	38.9 ± 10.1		49.3 ± 8.0	

Working experience as SHV	≤ 10 years	45.8 ± 7.6	0.03^**∗**^	56.6 ± 9.2	0.09
	11–20 years	39.4 ± 8.9^∗∗^		49.8 ± 7.4^∗∗^	
	≥ 21 years	42.0 ± 7.3		51.1 ± 4.4	

Administrative district	Nicosia	45.2 ± 9.2	0.02^**∗**^	48.6 ± 7.4	0.09
	Limassol/Paphos	38.6 ± 7.6^∗∗^		53.7 ± 8.3	
	Larnaca/Ammochostos	42.9 ± 9.3		55.2 ± 7.9^∗∗^	

^∗^ *p* ≤ 0.05 in the ANOVA test.

^∗∗^Post hoc Bonferroni tests indicated statistically significant differences among the groups at an alpha level of 0.05 assuming the first category as the reference.

**Table 7:** Univariable, multivariable and backward stepwise binary logistic regression analysis for the identification of support for school-based intervention program DDS events.

			Univariable analysis			Multivariable analysis			Backward LR stepwise		
			OR	95% CI	*p* value	OR	95% CI	*p* value	OR	95% CI	*p* value
Education level (graduate vs. postgraduate)			0.93	(0.37–2.34)	0.87	1.34	(0.35–5.16)	0.67			

Work experience as SHV	≤ 10 years		—	—	—	—	—	—			
	11–20 years		1.15	(0.38–3.50)	0.80	3.53	(0.67–18.70)	0.14			
	≥ 21 years		0.69	(0.11–4.24)	0.69	4.07	(0.22–73.95)	0.34			

DDS episodes may have possible adverse effects on children with asthma (more support vs. ref: less support)			7.60	(2.38–24.29)	< 0.001	17.65	(1.99–156.50)	0.01	7.33	(2.12–25.31)	< 0.001

Due to climate change, health problems associated with DDS episodes will increase among school-aged children (more support vs. ref: less support)			2.94	(1.07–8.10)	0.04	0.68	(0.13–3.68)	0.65			

Managing the impact of desert dust episodes on children's health is a priority for your service agree versus ref: disagree			0.82	(0.33–2.04)	0.66	0.51	(0.10–2.59)	0.41			

Score for school nurses' current practices on childhood asthma management		Poor	—	—	—	—	—	—			
		Satisfactory	1.64	(0.52–5.14)	0.40	1.63	(0.32–8.40)	0.56			
		Outstanding	1.75	(0.56–5.44)	0.34	2.96	(0.46–19.12)	0.25			

Score for current practices of SHV service to respond to DDS events		Poor	—	—	—	—	—	—			
		Satisfactory	1.35	(0.418–4.364)	0.62	2.27	(0.45–16.60)	0.27			
		Outstanding	0.40	(0.127–1.26)	0.11	0.12	(0.10–4.99)	0.71			

Abbreviations: CI, confidence interval; OR, odds ratio.

## Data Availability

The data that support the findings of this study are available from the corresponding author upon reasonable request.

## Supporting Information

The Supporting section includes the structured questionnaire used in this study translated in the English language.
