# Supplementary material for: Health Effects of Desert Dust Storms in Children With Asthma: Knowledge, Perceptions and Practices of School Health Visitors in Cyprus
Source: Nurs Res Pract. 2025 Apr 16;2025:8840328. doi: 10.1155/nrp/8840328 (PMC12017961; doi:10.1155/nrp/8840328)
Supplement: Supporting Information — Additional supporting information can be found online in the Supporting Information section. [file 8840328.f1.docx]

**Supplementary File 1 – Study Questionnaire**

**Part A- Demographics**

| Title: | - School Doctor | | - School Nurse |
| --- | --- | --- | --- |
| Province of Employment: | - Nicosia - Limassol - Larnaca/ Famagusta - Paphos | | |
| Gender: | - Male | | - Female |
| Age: | - <29 years - 30-39 years - 40-49 years | | |
|  | - 50-59 years | | |
|  | - >60 years | | |
| Level of Education: | - Higher Education Diploma - Bachelor’s Degree - Medical Specialty - Master’s Degree - Doctorate | | |
| Years of Service: | |  | |
| Years of service in the  community/ Health Service: | |  | |
| Field of Work  **Check all the answers that apply* | - Schools - Maternity and Child Welfare Clinics - Health care centers - Other…………………………… | | |
| Please indicate the number of students that you monitor this  school year (*if applicable) | - Primary School ………………. - Lower Secondary Education(Gymnasium)……………….. - Higher Secondary Education(Lyceum)……………….. | | |

**Part B**

***The table below lists some practices related to the management of childhood asthma by your***

***service.*There is no right or wrong answer. Please choose the answer which represents your opinion.**

| **Childhood Asthma Management** | | **Never** | **Rarely** | **Sometimes** | | **Often** | **Always** |
| --- | --- | --- | --- | --- | --- | --- | --- |
| **How frequently do you perform the following practices?** | | | | | | | |
|  | Assessment of students’ level of asthma control (clinical examination) during the school year | 1 | 2 | 3 | | 4 | 5 |
|  | Assessment of student’s asthma control (symptoms) and awareness of asthma triggers during the school year | 1 | 2 | 3 | | 4 | 5 |
|  | Contact health care provider to obtain investigations’ results (e.g. peak flow measurements) or management guidelines at the beginning of the school year | 1 | 2 | 3 | | 4 | 5 |
|  | Request updated student health records and monitoring of students’ immunization status | 1 | 2 | 3 | | 4 | 5 |
|  | Development of an asthma action plan for each student with asthma | 1 | 2 | 3 | | 4 | 5 |
|  | Provision of education to students to self-manage their asthma | 1 | 2 | 3 | | 4 | 5 |
|  | Assessment of implementation of asthma management recommendations by children with asthma and their family | 1 | 2 | 3 | | 4 | 5 |
|  | Provision of written instructions for asthma management during school hours | 1 | 2 | 3 | | 4 | 5 |
|  | Assessment of inhalers’ use technique of the students with asthma | 1 | 2 | 3 | | 4 | 5 |
|  | Tracking missed schooling days for asthma related symptoms in children with asthma | 1 | 2 | 3 | | 4 | 5 |
|  | Referring students to primary health care if their asthma is not under control | 1 | 2 | 3 | | 4 | 5 |
|  | Acquisition of information from school on children who are asthmatic as well as their medication status at the beginning of the school year | 1 | 2 | 3 | | 4 | 5 |
|  | Acquisition of information from parents of children with asthma for laboratory investigations results, medication and course of disease | 1 | 2 | 3 | | 4 | 5 |
|  | Acquisition of information from teachers about exacerbations in children with asthma | 1 | 2 | 3 | | 4 | 5 |
|  | Based on your own experience, which of the listed actions are the most important in the effective management of students with asthma in your school. Use a number between 1 to 10, with 1 referring to “not important” and 10 “absolutely important”. | | | | | | |
|  | Performance of regular check ups | | | |  | | |
|  | Evaluation of awareness of asthma symptoms/triggers | | | |  | | |
|  | Laboratory investigations requests | | | |  | | |
|  | Personalised action plan | | | |  | | |
|  | Asthma self-management education | | | |  | | |
|  | Communication with parents | | | |  | | |
|  | Asthma management education to school faculty | | | |  | | |
|  | Training/assessing inhaler technique | | | |  | | |
|  | Tracking of health records/immunization status of students | | | |  | | |
|  | Tracking of missed schooling days due to asthma exacerbation | | | |  | | |
|  | Communication with other health professionals | | | |  | | |
|  | Other:……………………………………………………………………………………………………. | | | |  | | |

**Part C**

***The table below lists some statements related to desert dust storm events***

**There is no right or wrong answer. Please choose the answer which represents your opinion.**

| **VIEWS ON DESERT DUST STORM EVENTS** | | **Strongly Disagree** | **Disagree** | **Neutral** | **Agree** | **Strongly**  **Agree** |
| --- | --- | --- | --- | --- | --- | --- |
| **To what extent do you agree or disagree with the following statements?** | | | | | | |
|  | DDS episodes may have possible adverse effects on children with asthma | 1 | 2 | 3 | 4 | 5 |
|  | Due to climate change, health problems associated with DDS episodes will increase among school-aged children. | 1 | 2 | 3 | 4 | 5 |
|  | Please indicate the extent to which you agree or disagree that the following stakeholders are aware of the potential adverse effects of DDS episodes on children with asthma | | | | | |
|  | School Health Service | 1 | 2 | 3 | 4 | 5 |
|  | Ministry of Health | 1 | 2 | 3 | 4 | 5 |
|  | Ministry of Education | 1 | 2 | 3 | 4 | 5 |
|  | Other Governmental bodies | 1 | 2 | 3 | 4 | 5 |
|  | Please indicate the extent to which you agree or disagree that the following stakeholders have sufficient experience to manage the potential health impacts of DDS on school-age children | | | | | |
|  | School Health Service | 1 | 2 | 3 | 4 | 5 |
|  | Ministry of Health | 1 | 2 | 3 | 4 | 5 |
|  | Ministry of Education | 1 | 2 | 3 | 4 | 5 |
|  | Other Governmental bodies | 1 | 2 | 3 | 4 | 5 |
|  | Please indicate the extent to which you agree or disagree that the following stakeholders have the expertise to create an effective plan for adapting to climate change and managing potential health impacts. | | | | | |
|  | School Health Service | 1 | 2 | 3 | 4 | 5 |
|  | Ministry of Health | 1 | 2 | 3 | 4 | 5 |
|  | Ministry of Education | 1 | 2 | 3 | 4 | 5 |
|  | Other Governmental bodies | 1 | 2 | 3 | 4 | 5 |
|  | Managing the impact of DDS episodes on children's health is a priority for your service. | 1 | 2 | 3 | 4 | 5 |

**Part D**

***The table below lists some practices related to desert dust storm events***

**There is no right or wrong answer. Please choose the answer which represents your opinion.**

| **Current Practices implemented by your service in relation to DDS** | | **Never** | **Rarely** | **Sometimes** | **Often** | **Always** |
| --- | --- | --- | --- | --- | --- | --- |
|  | Your Service receives warnings from a responsible government agency when dust storms occur | 1 | 2 | 3 | 4 | 5 |
|  | Your Service uses the “Air Quality Cyprus” application to be informed about the air quality throughout the year. | 1 | 2 | 3 | 4 | 5 |
|  | Your Service manages health issues due to desert dust episodes. | 1 | 2 | 3 | 4 | 5 |
|  | Instructions are provided by your Department for the management of asthma in children during desert dust episodes. | 1 | 2 | 3 | 4 | 5 |
|  | You receive training / information from your Service, through seminars, on new data on the management of childhood asthma during dust storms in the atmosphere. | 1 | 2 | 3 | 4 | 5 |
|  | During the school year, you keep record of all necessary information for all school children with asthma. | 1 | 2 | 3 | 4 | 5 |
|  | During DDS dust episodes, children with asthma are monitored by your service. | 1 | 2 | 3 | 4 | 5 |
|  | During the school year, you give lectures to both teachers and students on topics related to desert dust protection measures. | 1 | 2 | 3 | 4 | 5 |
|  | During the school year you disseminate information in the form of informational material with measures to protect from DDS. | 1 | 2 | 3 | 4 | 5 |

| **Practices related to DDS events implemented by other services** | | **Strongly Disagree** | **Disagree** | **Neutral** | **Agree** | **Strongly**  **Agree** |
| --- | --- | --- | --- | --- | --- | --- |
|  | You are aware of any additional desert dust prevention measures taken by other agencies (e.g. Ministry of Education). | 1 | 2 | 3 | 4 | 5 |
|  | You track missed schooling days of children with asthma due to their asthma exacerbation associated with DDS episodes. | 1 | 2 | 3 | 4 | 5 |
|  | You get informed for possible asthma exacerbation of children related to DDS episodes from the school stuff. | 1 | 2 | 3 | 4 | 5 |
|  | Your school is taking preventive measures issued by the Ministry of Education and Culture regarding DDS episodes. | 1 | 2 | 3 | 4 | 5 |
|  | The school implements and modifies its daily schedule during DDS episodes. | 1 | 2 | 3 | 4 | 5 |

**Part E**

***The table below lists potential practices for desert dust storm event management from your service. On a scale from 1 to 5, with 1 referring to “Not at all important” and 5 “Extremely important”,* please choose the answer which represents your opinion on the importance of each practice.**

| **Views on School based programs** | | **Not at all important** | **Slightly**  **Important** | **Moderately Important** | **Very**  **Important** | **Extremely Important** |
| --- | --- | --- | --- | --- | --- | --- |
| **How important is the following statements to you?** | | | | | | |
|  | The implementation of a school based program that will aim to reduce exposure to concentrations of desert dust particles. | 1 | 2 | 3 | 4 | 5 |
|  | The program should involve the education of the students | 1 | 2 | 3 | 4 | 5 |
|  | The program should involve the education of the parents | 1 | 2 | 3 | 4 | 5 |
|  | The program should involve the education of the teachers | 1 | 2 | 3 | 4 | 5 |
|  | The program should include guidelines for students to reduce exposure to desert dust during school hours (eg children should not to exercise outdoors on days with high concentrations of desert dust particles). | 1 | 2 | 3 | 4 | 5 |
|  | The program should include guidelines for students to reduce exposure to desert dust after school hours (e.g. closed windows, minimizing time outdoors and commuting). | 1 | 2 | 3 | 4 | 5 |
|  | The program should include instructions to the school to cancel any outdoor educational visits or school excursions during desert dust storm days | 1 | 2 | 3 | 4 | 5 |
|  | The program should include instructions to clean contact surfaces (e.g. desks, benches)with a wet cloth and mop the floor during desert dust storm days | 1 | 2 | 3 | 4 | 5 |
|  | The program should include instructions to the parents to avoid rigorous physical activity of their child during desert dust days | 1 | 2 | 3 | 4 | 5 |
|  | The program should include the instructions to avoid activities that may worsen indoor air quality such as using vacuum cleaners, cooking with combustible sources or gas, lighting candles and fireplaces and smoking cigarettes) | 1 | 2 | 3 | 4 | 5 |
|  | The program should include recommendations for children to wear face mask during desert dust days | 1 | 2 | 3 | 4 | 5 |
|  | The program should include recommendations for the use of an air cleaning devise( eg use of an air cleaner in the classrooms) | 1 | 2 | 3 | 4 | 5 |
|  | The program should include the development of audio-visual material with instructions to children, parents and teachers. | 1 | 2 | 3 | 4 | 5 |
|  | School Doctors and School health visitors should participate in the program | 1 | 2 | 3 | 4 | 5 |
|  | An interdisciplinary team should participate in the program. | 1 | 2 | 3 | 4 | 5 |

**Thank you for completing our survey.**
